# Geoffrey Commeline Williams

**Published:** 1949-10

**Authors:** 


### Obituaries

### GEOFFREY COMMELINE WILLIAMS

*O.B.E.*, M.A. Oxon. et Cantab., M.R.C.S. Eng., L.R.C.P. Lond., D.P.H. Bristol.

PERHAPS only some of our senior members have personal recollections of G. C. Williams, who died on September 21st, at the age of 57. The son of Dr. Williams of Thornbury, he was educated at Clifton College, at Gonville and Caius College, Cambridge, and at Bristol University. He qualified in 1916 and shortly after went overseas in the R.A.M.C.

On demobilization, Williams was appointed assistant M.O. at Ham Green Hospital and took the D.P.H. in 1921. Soon after this he was appointed assistant M.O.H. Oxford, where he spent the remainder of his life. He was appointed M.O.H. Oxford in 1930 and on the introduction of the National Health Service was made senior administrative M.O. to the Oxford Regional Hospitals Board. He was an active member of several societies concerned in public health administration and occupied important positions both in the University of Oxford and on the board of the Nuffield Institute. In 1936 he was Vice-President of the section of Public Health of the B.M.A. and in 1942 was awarded the O.B.E. Williams had a quiet and modest manner which might be mistakenly attributed to diffidence : the many important positions to which he was called in the course of a very busy and fruitful professional life testify to the esteem in which he was held by those who knew him best. We desire to offer our sympathies to his widow and daughter.

